# The Impact of Biofilm Formation on the Persistence of Candidemia

**DOI:** 10.3389/fmicb.2018.01196

**Published:** 2018-06-04

**Authors:** Wei-Sin Li, Yi-Chun Chen, Shu-Fang Kuo, Fang-Ju Chen, Chen-Hsiang Lee

**Affiliations:** ^1^Division of Infectious Diseases, Department of Internal Medicine, Kaohsiung Chang Gung Memorial Hospital, Kaohsiung, Taiwan; ^2^Department of Laboratory Medicine, Kaohsiung Chang Gung Memorial Hospital, Kaohsiung, Taiwan; ^3^Chang Gung University College of Medicine, Kaohsiung, Taiwan

**Keywords:** candidemia, biofilm, invasive candidiasis, antifungal susceptibility, vascular catheter

## Abstract

This study aimed to determine the predictors of persistent candidemia and examine the impact of biofilm formation by *Candida* isolates in adult patients with candidemia. Of the adult patients with candidemia in Kaohsiung Chang Gung Memorial Hospital between January 2007 and December 2012, 68 case patients with persistent candidemia (repeated candidemia after a 3-day systemic antifungal therapy) and 68 control patients with non-persistent candidemia (*Candida* clearance from the bloodstream after a 3-day systemic antifungal therapy) were included based on propensity score matching and matching for the *Candida* species isolated. Biofilm formation by the *Candida* species was assessed *in vitro* using standard biomass assays. Presence of central venous catheters (CVCs) at diagnosis (adjusted odd ratio [AOR], 3.77; 95% confidence interval [CI], 1.09–13.00, *p* = 0.04), infection with higher biofilm forming strains of *Candida* species (AOR, 8.03; 95% CI, 2.50–25.81; *p* < 0.01), and receipt of suboptimal fluconazole doses as initial therapy (AOR, 5.54; 95% CI, 1.53–20.10; *p* < 0.01) were independently associated with persistent candidemia. Biofilm formation by *Candida albicans*, *C. tropicalis*, and *C. glabrata* strains was significantly higher in the case patients than in the controls. There were no significant differences in the overall mortality and duration of hospitalization between the two groups. Our data suggest that, other than presence of retained CVCs and use of suboptimal doses of fluconazole, biofilm formation was highly associated with development of persistent candidemia.

## Introduction

The mortality among patients with invasive candidiasis is as high as 40%, which has been shown to be related to severity of the underlying disease, presence of retained infected vascular catheters, and delayed initiation of appropriate antifungal therapy (Kullberg and Arendrup, 2015; Pappas et al., 2016). To improve therapeutic strategies for candidemia, the most common manifestation of invasive candidiasis (Kullberg and Arendrup, 2015), the Infectious Disease Society of America (IDSA) treatment guidelines recommend follow-up blood cultures every day or every other day in patients with candidemia until clearance of *Candida* from the bloodstream (Pappas et al., 2016).

Persistent fungemia, an increasingly recognized complication of candidemia, has been reported in 8–15% of patients with candidemia (Nucci, 2011). In addition to host factors, such as poor general condition, several possible mechanisms have been suggested for persistent candidemia: drug resistance, low serum drug levels, endovascular infection, deep-tissue abscesses, and infections associated with prosthetics (Nucci, 2011). Unlike bacteremia, in which persistent positive blood cultures after initiation of antibiotic therapy indicates poor clinical outcome, persistent candidemia has not consistently been associated with increased mortality compared with non-persistent candidemia (Nucci, 2011). However, the case fatality rate among neonates with persistent candidemia was significantly higher than among those with non-persistent candidemia (Hammoud et al., 2013). Currently, the clinical significance of persistent candidemia remains debatable. The intensive care unit (ICU) stay, the presence of retained vascular catheters, and severity of illness were independent predictors of death (Nucci, 2011; Hammoud et al., 2013; Kang et al., 2017). In contrast, early mortality might preclude repetitive blood sampling from being performed for cultures during candidemia management, which may lead to immortal bias when examining the relationship between persistent candidemia and mortality. Well-designed studies with blood cultures performed in a timely fashion in the patients with candidemia are warranted to address this issue.

A biofilm is mainly composed of sessile cells and extracellular matrix, and its formation is initiated by the adherence of free-moving planktonic cells to surfaces of the human endothelium or medical implants (Donlan and Costerton, 2002). Biofilm formation is considered a virulence factor because it might lead to antifungal resistance and protection of fungal cells from immune responses. Biofilm formation by the *Candida* species may also be responsible for these fungi being well adapted to colonization of tissues and indwelling devices, which may lead to difficulties in eradicating these micro-organisms from the blood (Ramage et al., 2012; Taff et al., 2013). Biofilm formation is considered an independent predictor of mortality among patients with candidemia (Tumbarello et al., 2007). To our knowledge, little is known about the relationship between the microbiological characteristics of *Candida* isolates, such as biofilm formation, and the risk of persistent candidemia. Thus, this study aimed to identify the factors associated with persistent candidemia and to examine the impact of biofilm formation among adult patients with persistent candidemia.

## Materials and Methods

### Hospital Setting and Patients

This study was conducted at Kaohsiung Chang Gung Memorial Hospital, a 2,700-bed facility serving as a primary care and tertiary referral center in southern Taiwan, between January 2007 and December 2012. This retrospective study with a waiver of patient consent was approved by the Institutional Review Board of Chang Gung Memorial Hospital [No. 2016-01495B0].

Adult patients (aged ≥ 18 years) with candidemia who had blood cultures repeated after antifungal therapy had been continued for at least 3 days were included in this study. If patients experienced more than one candidemia episode, only the first episode was analyzed. All of the blood cultures were obtained directly from peripheral vessels of patients, not from any retained vessel catheters. The demographic and clinical information were retrieved, including age, gender, underlying diseases, laboratory data [neutrophil counts, albumin, and creatinine for estimated glomerular filtration rate (eGFR)], candidemia severity at onset [acute physiology and chronic health evaluation (APACHE) II score and the sequential organ failure assessment (SOFA) score], exposure to antibiotic and antifungal agents within 1 month before onset of candidemia, treatment regimens, and clinical outcomes. Patients’ outcomes included duration of antifungal treatment after onset of candidemia, early mortality, overall mortality, and duration of hospital stay.

### Definitions

Persistent candidemia was defined as presence of candidemia at repeat blood cultures in patients who had received systemic antifungal agents for at least 3 days (Safdar et al., 2004), and the *Candida* species identified from initial and repeat blood cultures were identical. The underlying diseases/conditions included type 2 diabetes mellitus (Handelsman et al., 2011), chronic kidney disease (eGFR < 60 mL/min per 1.73 m^2^) (Levey et al., 2003), liver cirrhosis (diagnosed by an abdominal sonography) (Hung et al., 2003), solid tumors, hematologic diseases, organ transplantation, and high-dose steroid use (≥0.3 mg/kg prednisolone or its equivalent for more than 3 weeks) (Lionakis and Kontoyiannis, 2003). Neutropenia was considered to occur when the granulocyte count was <1000 mm^3^ (Nucci and Colombo, 2002), and malnutrition was defined as albumin level of <3.0 g/dl. Recent chemotherapy and abdominal surgery were defined as receiving therapy within 1 month before onset of candidemia. Acute kidney injury was defined as a decreased eGFR below 50% of the normal range (Bellomo et al., 2004). Presence of a central venous catheter (CVC) at diagnosis of candidemia was recorded, and early CVC removal was defined as removing CVC within 48 h after detection of candidemia (Nucci et al., 2010). Shock was diagnosed as having a mean arterial pressure <65 mmHg, the occurrence of peripheral hypoperfusion, and the need for vasopressors (Rhodes et al., 2017). Infectious disease (ID) consultation within 48 h after blood culture collection referred to both the initial consultation and the period when patients were already followed by an ID specialist (Farmakiotis et al., 2015). Antifungal therapy was considered inappropriate when (i) *Candida* isolates were resistant to empirical antifungal therapy according to the CLSI M27-S4 document (Clinical and Laboratory Standards Institute [CLSI], 2012), (ii) the loading dose or/and maintenance dose of antifungal agents was insufficient (Gilbert et al., 2017), or/and (iii) the first dose of antifungal agents was used more than 48 h after positive blood cultures. The antimicrobial regimens and dosage were selected at the discretion of the attending physician. The early and overall mortality were defined as death from any cause within 14 days from the onset of candidemia and during hospitalization, respectively.

### Identification and Antifungal Susceptibility

Microorganisms isolated from the blood cultures were detected using the BD BACTEC FX system (Becton, Dickinson Microbiology System, Sparks, MD, United States). These cultures were incubated for 5 days at 35°C. A yeast-like microorganism was detected by microscopic inspection after Gram staining. A subsequent identification of *Candida* spp. was performed with the use of CHROMagar *Candida* (Bectons, Dickson and Company Co.) or API-ID32C (BioMerieux, Vitek, Hazelwood, MO, United States). *Candida* isolates were stored at -70°C in a skim milk with glycerol until they were tested (Diagnostic Systems, Sparks, MD, United States).

The antifungal susceptibility of the isolates was classified in accordance with the Clinical and Laboratory Standards Institute (CLSI) M27-S4 document (Clinical and Laboratory Standards Institute [CLSI], 2012). For antifungal susceptibility testing, all isolates were thawed and inoculated onto Sabouraud dextrose agars (SDA) and incubated at 35°C for 24 h. Subculture from SDA was performed to obtain a pure 24-h culture of the *Candida* isolate and to prepare a suspension with an organism density of approximately 1.5 × 10^3^ CFU/mL. The susceptibility of all tested isolates to six antifungal agents (amphotericin B, fluconazole, voriconazole, anidulafungin, caspofungin, and micafungin) was determined using the broth microdilution method with the Sensititre YeastOne (SYO) system (part YO-09; Trek Diagnostic System) according to the manufacturer’s instructions. The broth microdilution method with SYO system was formed in a 96-well microtiter plate and prepared with 12 dilutions of each drug: amphotericin B concentration ranges from 0.12 to 4 mg/L, fluconazole0.12 to 256 mg/L, voriconazole 0.008 to 8 mg/L, anidulafungin 0.015 to 8 mg/L, micafungin 0.008 to 8 mg/L, and caspofungin 0.008 to 8 mg/L. Plates were inoculated using a prepared suspension of the organism. Alamar Blue was incorporated in each well, which displayed a change in color from blue to pink in the presence of a microbial growth. Colorimetric MIC results for all agents tested were defined as the lowest concentration of antifungal agent that prevented the development of red colonies. Quality control was performed by testing the CLSI recommended strains, *C. krusei* ATCC 6258, and *C. parapsilosis* ATCC 22019.

### Biofilm Testing and Antifungal Susceptibility on Biofilm Formation of *C. albicans*

*Candida* isolates displayed heterogeneity with respect to their biofilm biomass when grown in RPMI. Standardized *Candida* isolates in RPMI-1640 were grown in flat-bottomed 96-well microtiter plates for 24 h at 37°C. Mature biofilms were carefully washed with PBS, allowed to air dry, and biomass quantified by staining with 0.05% w/v crystal violet (CV) solution. The biofilms were washed and distained with 100% ethanol. The biomass was quantified spectrophotometrically by reading the absorbance at 550 nm in a microtiter plate reader (FluoStar Omega BMG Labtech, Aylesbury, United Kingdom). Three replicates were used for each isolate and were carried out on one occasion. Isolates of each species were categorized as low biofilm formers (LBFs) or high biofilm formers (HBFs) if their biomass absorbance values were less than the first quartile (Q_1_) or greater than the third quartile (Q_3_), respectively. Those isolates in between the first and third quartiles (Q_1_–Q_3_) were defined as intermediate biofilm formers (IBF) (Sherry et al., 2014).

Furthermore, the *C. albicans’* biofilm formation to different classes of antifungal agents was also assessed. The biofilms were treated with either 4 mg/L or 256 mg/L fluconazole, caspofungin, or amphotericin B. After incubation for 24 h, the metabolic activity was measured with the XTT assay, with the optical density being read at 492 nm (Pierce et al., 2008). The percentage viability was calculated relative to the untreated controls, and the data were presented as mean ± standard deviation. Three replicates were used for each isolate and were carried out on one occasion.

### Testing for Invasiveness of *Candida* Isolates

*Candida* strains were streaked out with a flat toothpick to form single colonies (typically 50 colonies/plate) on YPD plates (yeast extract “Difco” 2%, Bacto Peptone “Difco” 4%, glucose 2%, Bacto-agar “Difco” 2%, and tryptophan 0.003%). All plates were incubated at 37°C, and the extent of invasion was scored after 2 days. Invasion was determined as follows: (i) cells that had not invaded the agar were washed away by rubbing the plate with a gloved finger while rinsing under running water, and (ii) cells that had invaded the agar remained as macroscopically visible microcolonies on the surface of the washed plate and confirmed by a microscopical examination (with a bright light field, 10× magnification) after washing. The invasiveness of *Candida* isolation was defined as follows: (i) strong invasiveness: more than two-thirds of the colonies kept retained in the agar after washing, (ii) moderate invasiveness: one-third to two-thirds of the colonies kept retained in the agar after washing, and (iii) weakness invasiveness: less than one-third of the colonies kept retained in the agar after washing (Supplementary Figure S1) (Kontoyiannis et al., 2001).

### Statistical Analysis

The included patients with candidemia were divided into two groups for analyses: patients with and those without persistent candidemia. Furthermore, the propensity score described the probability, on the basis of age, gender, underlying disease, and disease severity. Subsequently, patients with and those without persistent candidemia were matched (1:1) by propensity score with a caliper radius of 0.2 sigma, *Candida* species (forced match variable), APACHE II score, and SOFA score (covariate variables) with the use of NCSS (Version 11, LLC, Kaysville, UT). Dichotomous variables were analyzed with a *x*^2^ test or Fisher’s exact test appropriately, and continuous variables were analyzed using the Mann–Whitney *U*-test. The variables with a *p*-value ≤ 0.2 in univariate analysis were then included in the forward stepwise conditional logistic regression analysis. The discriminatory power of this derivation model was tested using the receiver operating curve (ROC) analysis by assessing the area under the curve (AUC), and the calibration efficiency was tested using the Hosmer–Lemeshow goodness-of-fit test for estimating goodness of fits to the data. A *p*-value ≤ 0.05 was considered statistically significant. A statistical analysis was conducted using the SPSS statistical analysis system (Version 22.0, IBM Corp, Armonk, NY, United States).

## Results

**FIGURE 1:**
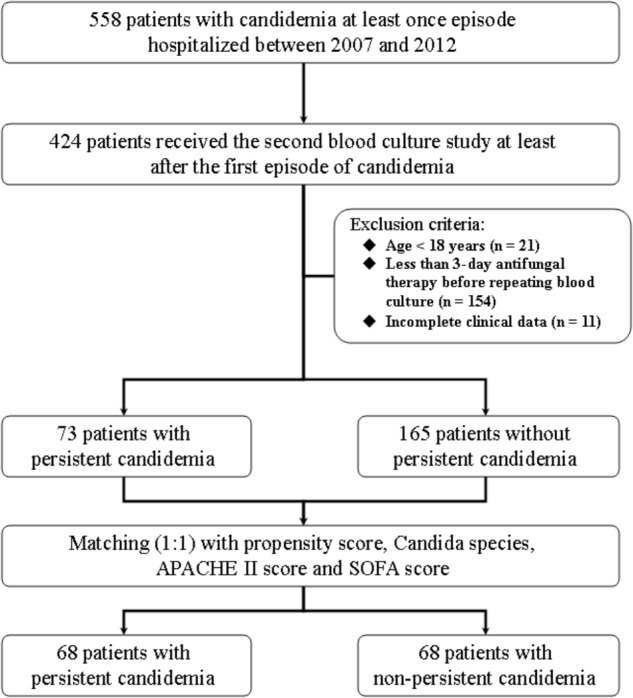
Patient selection flowchart. APACHE, acute physiology and chronic health evaluation; SOFA, sequential organ failure assessment.

A total of 558 adult patients with candidemia were hospitalized between 2007 and 2012, and 424 (76.0%) had blood cultures repeated after the first growth of *Candida* spp. Patients aged <18 years (*n* = 21), those with less than 3-day antifungal therapy before repeating blood cultures (*n* = 154), and those with incomplete clinical data (*n* = 11) were excluded. As a result, 238 patients were eligible for case-control study, including 73 with persistent candidemia. Among these 73 patients with persistent candidemia, 33 were infected by *C. albicans*, 18 by *C. tropicalis*, 11 by *C. parapsilosis*, 10 by *C. glabrata*, and 1 by *C. krusei*. To reduce the bias of some cases in which blood cultures were not obtained in a timely fashion, we matched 68 patients with persistent candidemia (case patients) with 68 patients without persistent candidemia (control patients) by propensity score, *Candida* species, APACHE II, and SOFA score (**Figure 1**). The standardized difference was 42.85% before propensity-score matching and was 1.59% after matching.

After propensity-score matching, there were no significant differences in terms of age, gender, underlying disease, disease severity, usage of total parenteral nutrition, concomitant bacteremia, and recent exposure to antibiotics, echinocandins, or azoles between the case patients and controls (**Table 1**). Case patients were more likely to have CVCs at the onset of candidemia but it did not reach significant difference (86.8% vs. 73.5%, *p* = 0.06). In the univariate analysis, case patients were less likely to receive empirical therapy with echinocandin (26.5% vs. 44.1%, *p* = 0.03), more received inappropriate antifungal therapy within 48 h after blood culture collection (64.7% vs. 41.2%, *p* = 0.01), including ineffective use of antifungal agents due to resistance (13.2% vs. 2.9%, *p* = 0.01) and suboptimal dose of antifungal agents (33.8% vs. 7.4%, *p* < 0.01). The antifungal agents with suboptimal dose all were prescribing fluconazole. There was no significant distinction in ID consultation within 48 h after blood culture collection, early CVC removal and early and overall mortality rates between case and control patients (**Table 2**).

**Table 1:** Characteristics of the 136 adult patients with persistent and non-persistent candidemia after propensity-score matching.

Characteristic	Persistent *N* = 68 (%)	Non-persistent *N* = 68 (%)	*P-*value
Age (year, range)	63 (30-89)	65 (26-86)	0.25
Gender, male	44 (64.7)	38 (55.9)	0.20
**Underlying disease**
Diabetes mellitus	24 (35.3)	26 (38.2)	0.67
Chronic renal disease	30 (44.1)	26 (38.2)	0.48
Liver cirrhosis	5 (7.4)	7 (10.3)	0.56
Solid tumor	25 (36.8)	25 (36.8)	>0.99
Leukemia	2 (2.9)	3 (4.4)	0.66
Lymphoma/myeloma	2 (2.9)	4 (5.9)	0.41
Solid-organ transplantation		1 (1.5)	0.32
Malnutrition (albumin < 3.0 g/dl)	42 (62.8)	39 (57.4)	0.59
Neutropenia (Neutrophil < 1,000 cells/μl)	4 (5.9)	4 (5.9)	>0.99
Receipt of corticosteroids (≥0.3 mg/kg prednisolone equivalent for ≥3 weeks)	3 (4.4)	5 (7.4)	0.48
Recent chemotherapy (within 1 month before onset of candidemia)	10 (14.7)	11 (16.2)	0.82
Recent abdominal surgery (within 1 month before onset of candidemia)	10 (14.7)	10 (14.7)	>0.99
**Clinical characteristics at onset of candidemia**
APACHE II score (range)	19 (6-36)	21 (8-34)	0.70
APACHE II score > 20	31 (45.6)	34 (50.0)	0.32
SOFA score (range)	6 (0-14)	7 (0-16)	0.76
Hospital-onset infection	64 (94.1)	64 (94.1)	>0.99
ICU-onset infection	31 (48.4)	29 (45.3)	0.72
Total parenteral nutrition	15 (22.1)	13 (19.1)	0.66
Presence of CVCs at diagnosis	59 (86.8)	50 (73.5)	0.06
Acute kidney injury (≥50% decrease in eGFR from baseline)	23 (33.8)	26 (38.2)	0.56
Shock at presentation	19 (27.9)	22 (32.4)	0.51
Concomitant bacteremia	13 (19.1)	16 (23.5)	0.49
**Recent exposures of antimicrobial agents (within 1 month before onset of candidemia)**
Antibiotics	62 (92.2)	62 (92.2)	>0.99
Azoles	6 (8.8)	2 (2.9)	0.16
Echinocandins	1 (1.5)		0.61


*APACHE, acute physiology and chronic health evaluation; CVCs, central venous catheters; eGFR, estimated glomerular filtration rate; ICU, intensive care unit; SOFA, sequential organ failure assessment.*

**Table 2:** Treatment regimens and outcomes of the 136 adult patients with persistent and non-persistent candidemia after propensity-score matching.

Variable	Persistent *N* = 68 (%)	Non-persistent *N* = 68 (%)	*P-*value
**Regimens of antifungal agents**			
Empirical therapy with azoles	48 (70.6)	37 (54.4)	0.06
Empirical therapy with echinocandins	18 (26.5)	30 (44.1)	0.03
Empirical therapy with amphotericin B	2 (2.9)	1 (1.5)	0.56
Days of the first dose after culture (range)	2 (1–11)	2 (1–11)	0.82
Inappropriate treatment within 48h after blood culture collection	44 (64.7)	28 (41.2)	0.01
Ineffective antifungal agent use	9 (13.2)	2 (2.9)	0.01
Suboptimal fluconazole dosage	23 (33.8)	5 (7.4)	<0.01
Delayed antifungal therapy (first dose > 48 h after blood culture collection)	24 (35.3)	25 (36.8)	0.86
ID consultant within 48h after blood culture collection	20 (29.4)	25 (36.8)	0.37
Early CVC removal	20 (33.9)	16 (32.0)	0.62
**Outcomes**			
Days of antifungal treatment (range)	22 (5–61)	18 (2–55)	0.15
Early mortality (within 14 days after onset of candidemia)	16 (23.5)	10 (14.7)	0.18
Overall mortality	45 (66.2)	35 (51.4)	0.10
Days of hospitalization (range)	61 (11–400)	62 (13–230)	0.48


*CVCs, central venous catheters; ID, infectious disease physicians.*

**FIGURE 2:**
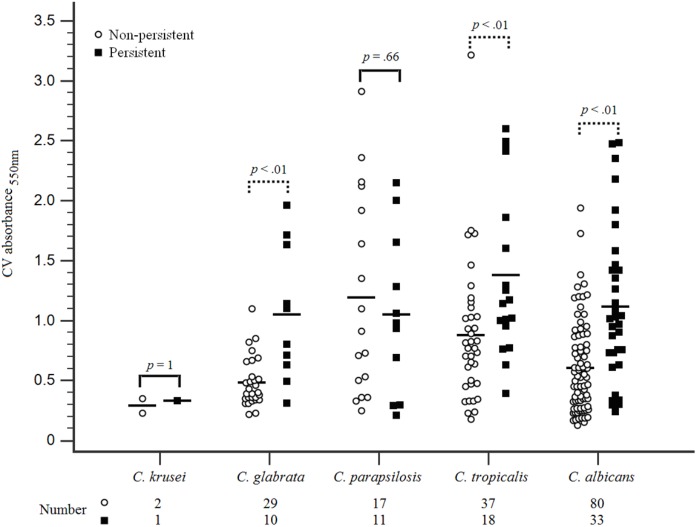
Biofilm formation on *Candida* species isolated from patients with persistent and non-persistent candidemia. *Candida* bloodstream strains isolated from all included patients were evaluated for biofilm formation with standardized methods. The first *Candida* spp. isolate was conducted for biofilm formation evaluation when multiple isolates were collected from some of these patients within the study period. *Candida* isolates standardized (1 × 10^6^ cells/mL) in RPMI-1640 were grown in flat-bottomed 96-well microtiter plates for 24 h at 37°C. The biomass quantified by staining with 0.05% w/v crystal violet solution. Biomass was quantified spectrophotometrically by reading the optical density (OD) at 550 nm in a microtiter plate reader (FluoStar Omega BMG Labtech, Aylesbury, United Kingdom). Three replicates were used for each isolate. The outlier was abandoned and the average of the 2 remaining values was represented. Higher biofilm formation in *C. albicans*, *C. tropicalis*, and *C. glabrata* were found among isolates in the persistent candidemia group compared with those in the non-persistent candidemia group.

There was no difference in the resistance to fluconazole or caspofungin among the *Candida* strains in both groups. The susceptibility of the initial and subsequent blood isolates of *Candida* species to antifungal agents in each case patient was determined, which revealed that the MICs of antifungal agents for the initial and subsequent isolates remained unchanged. The proportions of HBF and high-IBF were significantly higher among *Candida* strains isolated from the case patients than those isolated from controls (60.3% vs. 22.1%, *p* < 0.01; and 92.6% vs. 75.0%, *p* < 0.01, respectively) (**Table 3**). The levels of biofilm formation quantified by the CV assay for the 136 *Candida* isolates were listed in Supplementary Table S1. Of all *Candida* isolates identified (*n* = 238), we found that biofilm formation in the isolates of *C. albicans*, *C. tropicalis*, and *C. glabrata* from the case patients were higher than those from the controls (**Figure 2**).

**Table 3:** The microbiological characteristics of *Candida* strains isolated from the 136 adult patients with persistent and non-persistent candidemia after propensity-score matching.

Variable	Persistent *N* = 68 (%)	Non-persistent *N* = 68 (%)	*P-*value
***Candida* species**			
*C. albicans*	33 (48.5)	33 (48.5)	>0.99
*C. tropicalis*	17 (25.0)	17 (25.0)	>0.99
*C. parapsilosis*	8 (11.8)	8 (11.8)	>0.99
*C. glabrata*	9 (13.2)	9 (13.2)	>0.99
*C. krusei*	1 (1.5)	1 (1.5)	>0.99
**Resistance of antifungal agents**			
Resistance to fluconazole	14 (20.6)	13 (19.1)	0.71
Resistance to caspofungin	1 (1.5)	3 (4.4)	0.32
**Biofilm former**			
High biofilm former	41 (60.3)	15 (22.1)	<0.01
High and intermediate biofilm former	63 (92.6)	51 (75.0)	<0.01
Invasiveness
Strong and intermediate	22 (32.4)	27 (39.7)	0.37


**FIGURE 3:**
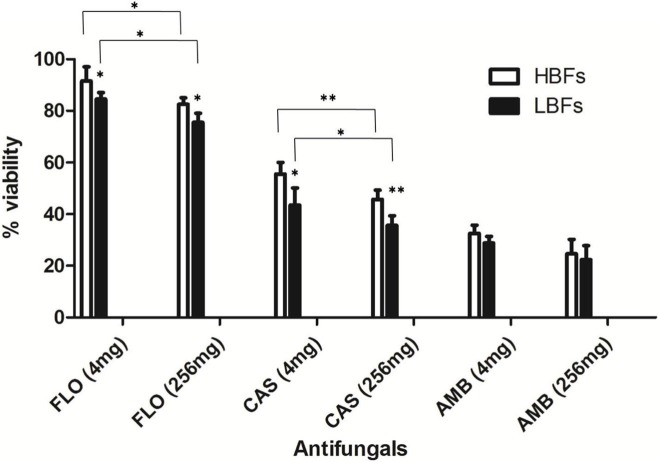
Impact of *Candida albicans* biofilm formation on antifungal susceptibility. Ten low biofilm formers (LBFs) and high biofilm formers (HBFs) were standardized to 1 × 10^6^ cells/mL in RPMI-1640, and grown as biofilms in flat-bottomed 96-well microtiter plates for 24 h. Biofilms were washed with phosphate-buffered saline before being treated with 4 mg/L or 256 mg/L fluconazole (FLO), caspofungin (CAS), and amphotericin B (AMB). Following treatment, the proportional viability was compared with that untreated control by use of an XTT metabolic assay. ^∗^*p* < 0.05, ^∗∗^*p* < 0.01.

The *C. albicans* isolates of LBFs (*n* = 10) and HBFs (*n* = 10) were tested for their susceptibility to azoles (fluconazole), polyenes (amphotericin B), and echinocandins (caspofungin) at low (4 mg/L) and high (256 mg/L) concentrations. Although fluconazole at a concentration of 4 and 256 mg/L were equally ineffective against mature HBFs and LBFs biofilms (**Figure 3**), a significant difference in the overall activity was observed between HBFs and LBFs at both 4 and 256 mg/L fluconazole (*p* < 0.05). Conversely, caspofungin and amphotericin B were effective against both HBFs and LBFs, although the levels of biofilm formation significantly impacted on the effectiveness of caspofungin between HBFs and LBFs (4 mg/L, *p* < 0.05; 256 mg/L, *p* < 0.01). Amphotericin B was shown to be equally effective against LBFs and HBFs, and no significant difference was found between HBFs and LBFs at 4 mg/L or 256 mg/L concentration. Furthermore, a 4 mg/L concentration of fluconazole and caspofungin was significantly less effective against both LBFs and HBFs than a 256 mg/L concentration of fluconazole and caspofungin.

**FIGURE 4:**
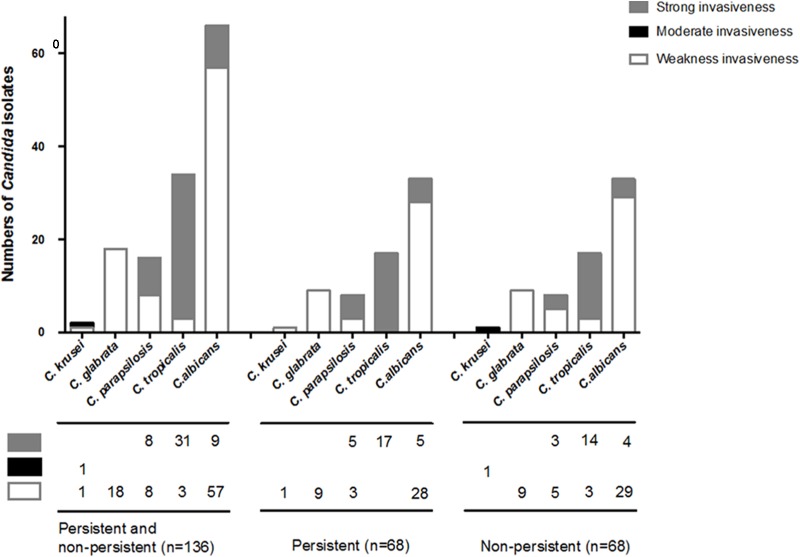
Invasiveness on *Candida* species isolated from persistent and non-persistent candidemia. Invasiveness was tested on 136 *Candida* strains isolated from including patients after propensity-score matching. *Candida* strains were streaked out with a flat toothpick to form single colonies (typically 50 colonies/plate) on YPD plates (yeast extract “Difco” 2%, Bacto Peptone “Difco” 4%, glucose 2%, Bacto-agar “Difco” 2%, and tryptophan 0.003%). All plates were incubated at 37°C and the extent of invasion was scored after 2 days. Invasion was determined as (i) cells that had not invaded the agar were washed away by rubbing the plate with a gloves finger while rinsing under running water, (ii) cells that had invaded the agar remained as macroscopically visible micro-colonies on the surface of the washed plate, and confirmed by micro-scopical examination (with a bright light field, 10× magnification) after washing. *Candida* strains with strong/intermediate invasiveness were not significantly different among the isolates from the patients between persistent and non-persistent group. In the 136 *Candida* strains, *C. tropicalis* (31/34; 91.2%) was the highest proportion of strains with strong invasiveness, followed by *C. parapsilosis* (8/16; 50%) and *C. albicans* (9/66; 13.6%).

Invasiveness was tested on 136 *Candida* strains isolated from the two groups of patients after propensity-score matching. With respect to the differences between biofilm formers, strains with strong/intermediate invasiveness were not significantly different among the isolates from the case patients and controls (32.4% vs. 39.7%, *p* = 0.37) (**Table 3**), even if classified by each *Candida* species (**Figure 4**). Of the 136 *Candida* strains (**Figure 4**), *C. tropicalis* (31/34; 91.2%) accounted for the highest proportion of isolates with strong invasiveness, followed by *C. parapsilosis* (8/16; 50%) and *C. albicans* (9/66; 13.6%).

A multivariate analysis showed that *Candida* isolates with HBFs (*p* < 0.01; odd ratio [OR] = 8.03; 95% confidence interval [CI] 2.50–25.81), initial treatment with suboptimal dosage of fluconazole (*p* < 0.01; OR = 5.54; 95% CI 1.53–20.10), and presence of CVCs at diagnosis of candidemia (*p* = 0.04; OR = 3.77; 95% CI 1.09–13.00) were the independent factors associated with persistent candidemia (**Table 4**). There was adequate goodness of fit (Hosmer–Lemeshow test, χ^2^ = 2.74, *p* = 0.25). The receiver operating characteristic analysis indicated that the predictive performance of logistic regression model was adequate (area under the curve = 0.76).

**Table 4:** Multivariate logistic regression analysis for independent factors of persistent candidemia.

Variable	Univariate	Multivariate	OR (95% CI)
Presence of CVCs at diagnosis	0.06	0.04	3.77 (1.09–13.00)
High biofilm former	<0.01	<0.01	8.03 (2.50–25.81)
High and intermediate biofilm former	<0.01	0.38	
Exposure of azoles	0.16	0.13	
Empirical therapy with azoles	0.06	0.41	
Empirical therapy with echinocandins	0.03	0.27	
Inappropriate treatment within 48h after blood culture collection	0.08	0.83	
Ineffective antifungal agent use	0.01	0.48	
Suboptimal fluconazole dosage	<0.01	<0.01	5.54 (1.53–20.10)


*CI, confidence interval; CVC, central venous catheter; OR, odds ratio.*

## Discussion

By comparisons of the clinical characteristics, microbiological features, and outcomes between patients with and those without persistent candidemia, we found that infection by *Candida* species with HBFs, presence of CVCs, and initial treatment with suboptimal dose of fluconazole were independently associated with persistent candidemia. Other than the presence of CVCs as a risk factor of persistent candidemia demonstrated in previous studies (Chen et al., 2012; Kang et al., 2017), our study also supports the current concept that biofilms formation are very important virulence factors for the establishment of recurrent candidiasis (Cuellar-Cruz et al., 2012) by directly demonstrating that HBFs are highly related to persistent candidemia. However, in the present study, we could not identify a beneficial effect of early CVC removal on prevention of persistent candidemia. A subgroup analysis of 2 randomized controlled trials also suggested that early CVC removal within 24 h or 48 h had no effect on persistent or recurrent candidemia (Nucci et al., 2010). In the retrospective study, blood cultures might not be obtained in a timely fashion to precisely establish the time point at which candidemia has been cleared. Understanding of the role of early CVC removal on persistent candidemia should rely on prospective studies with serial blood cultures performed at predefined intervals.

As already reported (Kang et al., 2017) and shown here, there were no serial MIC changes in the repeatedly cultured *Candida* strains from patients with persistent candidemia. While the early and overall mortality rates were not significantly different between the patients with and those without persistent candidemia after propensity-score matching that took into consideration the underlying diseases and severity at the onset of candidemia. Our findings illustrate that use of suboptimal dose of fluconazole was significantly higher among patients with persistent candidemia. It has been reported that suboptimal dosage of fluconazole was associated with poor response to treatment, defined as non-clearance of *Candida* species from bloodstream and mortality on day 7 after the onset of candidemia (Brosh-Nissimov and Ben-Ami, 2015). Therefore, pharmacodynamic-directed fluconazole dosing may help optimize outcomes for patients with candidemia.

Among the 238 *Candida* isolates (**Figure 2**), *C. parapsilosis* (11/28; 39.3%) was the most common pathogen associated with persistent candidemia, followed by *C. tropicalis* (18/55; 32.7%). We found that HBFs in *C. albicans*, *C. tropicalis*, and *C. glabrata* were higher in the patients with persistent candidemia than those without persistent candidemia, but the phenomenon was not shown in *C. parapsilosis* isolates. The biofilm-forming ability, structure, and matrix composition are highly species dependent with an additional strain variability occurring with *C. parapsilosis* (Silva et al., 2009). The *C. parapsilosis* complex is composed of three genetically distinct species, namely *C. parapsilosis* sensu stricto, *C. orthopsilosis* and *C. metapsilosis*, which are physiologically and morphologically indistinguishable (Tavanti et al., 2005). Previous data have shown that these three species exhibit different prevalence rates, virulence, and *in vitro* antifungal susceptibility. The species with the highest biofilm production was *C. parapsilosis* sensu stricto, followed by *C. orthopsilosis* and further by *C. metapsilosis* (Neji et al., 2017). However, in the present study, three genomic species of the *C. parapsilosis* species complex were not performed to classify which might contribute to the finding of no significant difference observed of biofilm formation in current *C. parapsilosis* isolates between case and control group.

We observed that caspofungin and amphotericin B rather than fluconazole were more effective against *C. albicans* with HBFs and LBFs. The viability of *C. albicans* was significantly decreased in high concentrations of fluconazole and caspofungin in comparison with exposure to low concentrations. However, amphotericin B is equally effective against both HBFs and LBFs groups regardless of the drug concentrations. The observation is in line with that of a previous report (Rajendran et al., 2016). It is noteworthy that persisters in *Candida* biofilms, the mean survivors after 24 h of treatment with high concentration of antifungal agents such as fluconazole, may have the capacity to invoke a specific adaptive strategy against anti-fungals (Li et al., 2015). Biofilm recalcitrance partially accounts for drug-tolerant persisters (Li et al., 2015). It was well known that echinocandins and lipid polyenes possess more activity against biofilm yeasts than do azoles (Uppuluri et al., 2011). In univariate analysis of the present study, patients without persistent candidemia were more likely to receive empirical antifungal with echinocandins than those with persistent candidemia. It was also demonstrated that, among the patients with biofilm-forming *Candida* bloodstream infection, those treated with highly active anti-biofilm agents (e.g., caspofungin) had significantly shorter hospital length of stay post-*Candida* bloodstream infection than those treated with non-highly active anti-biofilm agents antifungal agents (e.g., fluconazole) (Tumbarello et al., 2012).

The ability to generate the filamentous form of *Candida* is important for tissue invasion which is dependent upon species and influenced by environment. We found that *C. tropicalis* was the highest proportion of strains with strong invasiveness, followed by *C. parapsilosis.* The finding was consistent with previous study. *C. tropicalis* showed more extensive colonization and invasion in reconstituted human oral epithelium than *C. parapsilosis* and *C. glabrata* (Silva et al., 2011). In our study, the grade of invasiveness behaviors of *Candida* isolates did not affect the occurrence of persistent candidemia. It was revealed that biofilm formation might be more influential than invasiveness on the development of persistent candidemia.

Our study still has several limitations. It is a retrospective study. Clinical characteristics of the two groups of patients might not be completely captured in the medical records. The follow-up blood cultures in some cases were not obtained timely based on the recommendation of the IDSA to establish the time point at which candidemia has been cleared. However, we used a propensity score to match the disease severity between the two study groups with follow-up blood cultures, which may help alleviate the concerns about immortal bias. In addition, we did not perform further analysis to classify three genomic species of the *C. parapsilosis* complex. *C. parapsilosis* strains are heterogeneous in terms of the level of biofilm formation with a 1301-fold difference between the highest and lowest biofilm-producing strains while *C. orthopsilosis* and *C. metapsilosis* strains exhibit a more homogeneous behavior (Neji et al., 2017). Therefore, we cannot demonstrate if there existed difference in biofilm formation between case and control group caused by *C. parapsilosis* after distinguishing the three species. Finally, testing for invasiveness of *Candida* isolates was only performed in agar plates. A further *ex vivo* model to assess the invasiveness is needed to confirm the findings of this *in vitro* method.

In summary, HBFs, presence of CVCs at diagnosis, and initial treatment with suboptimal dose of fluconazole were independent factors associated with persistent candidemia. Using an adequate dose of fluconazole to maximize the effectiveness or use of highly active anti-biofilm agents, such as echinocandin or amphotericin B are strategies that warrant more investigations to prevent persistent candidemia.

## Author Contributions

W-SL, Y-CC, and C-HL conceptualized and designed the study, and drafted the manuscript. C-HL assisted with acquiring funding. W-SL collected clinical data. S-FK and F-JC conducted laboratory analyses. Y-CC and C-HL reviewed and validated the laboratory analysis. W-SL conducted the statistical analysis. All authors assisted with critically reviewing the manuscript.

## Conflict of Interest Statement

The authors declare that the research was conducted in the absence of any commercial or financial relationships that could be construed as a potential conflict of interest.
