# Supplementary material for: The Impact of Biofilm Formation on the Persistence of Candidemia
Source: Front Microbiol. 2018 Jun 4;9:1196. doi: 10.3389/fmicb.2018.01196 (PMC5994545; doi:10.3389/fmicb.2018.01196)
Supplement: Supplementary file 1 [file Image_1.pdf]

Supplementary Figure S1.

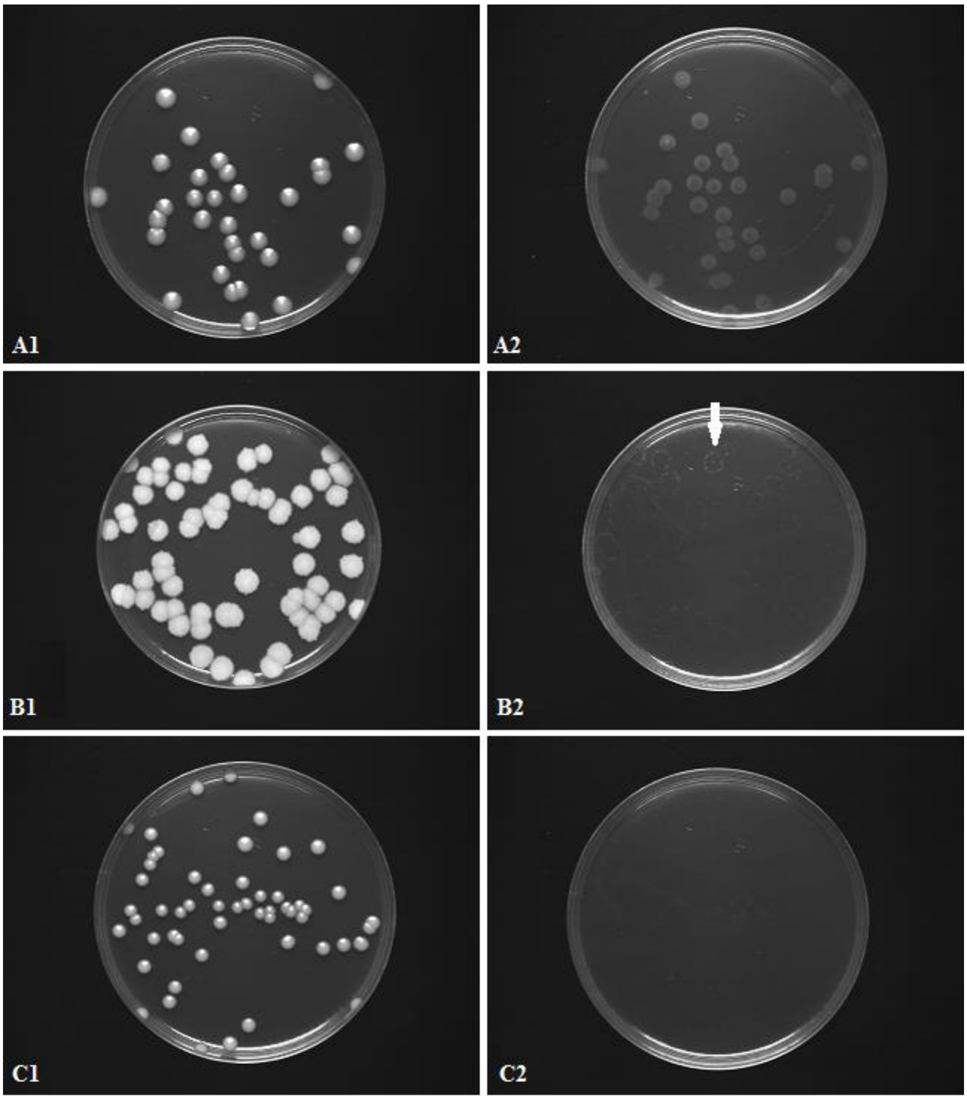

Supplementary Figure S1. Invasiveness on *Candida* species was defined as (i) strong invasiveness: more than two-third of colonies keep retention in the agar after washing (A1 and A2, before and after washing, respectively), (ii) moderate invasiveness: one-third to two-third of colonies keep retention in the agar (arrow) after washing (B1 and B2, before and after washing, respectively), and (iii) weak invasiveness, less than one-third of colonies in the agar keep retention in the agar after

washing (C1 and C2, before and after washing, respectively). Photography was taken with FloGel (FGIS-2 Fluorescent Gel Image System).
