# Supplementary material for: The Impact of Biofilm Formation on the Persistence of Candidemia
Source: Front Microbiol. 2018 Jun 4;9:1196. doi: 10.3389/fmicb.2018.01196 (PMC5994545; doi:10.3389/fmicb.2018.01196)
Supplement: Supplementary file 2 [file Table_1.DOC]

**Table S1**. Biofilm formation by 136 *Candida* isolates from 68 persistent and 68 non-persistent candidemic patients as assayed by crystal violet assay.

| No. of isolate | Species of *Candida* | Testing assay | | | |
| --- | --- | --- | --- | --- | --- |
| persistent group |  | Crystal violet | | | Average* |
| A1  A2  A3  A4  A5  A6  A7  A8  A9  A10  A11  A12  A13  A14  A15  A16  A17  A18  A19  A20  A21  A22  A23  A24  A25  A26  A27  A28  A29  A30  A31  A32  A33  T1  T2  T3  T4  T5  T6  T7  T8  T9  T10  T11  T12  T13  T14  T15  T16  T17  P1  P2  P3  P4  P5  P6  P7  P8  G1  G2  G3  G4  G5  G6  G7  G8  G9  K1 | *C. albicans*  *C. albicans*  *C. albicans*  *C. albicans*  *C. albicans*  *C. albicans*  *C. albicans*  *C. albicans*  *C. albicans*  *C. albicans*  *C. albicans*  *C. albicans*  *C. albicans*  *C. albicans*  *C. albicans*  *C. albicans*  *C. albicans*  *C. albicans*  *C. albicans*  *C. albicans*  *C. albicans*  *C. albicans*  *C. albicans*  *C. albicans*  *C. albicans*  *C. albicans*  *C. albicans*  *C. albicans*  *C. albicans*  *C. albicans*  *C. albicans*  *C. albicans*  *C. albicans*  *C. tropicalis*  *C. tropicalis*  *C. tropicalis*  *C. tropicalis*  *C. tropicalis*  *C. tropicalis*  *C. tropicalis*  *C. tropicalis*  *C. tropicalis*  *C. tropicalis*  *C. tropicalis*  *C. tropicalis*  *C. tropicalis*  *C. tropicalis*  *C. tropicalis*  *C. tropicalis*  *C. tropicalis*  *C. parapsilosis*  *C. parapsilosis*  *C. parapsilosis*  *C. parapsilosis*  *C. parapsilosis*  *C. parapsilosis*  *C. parapsilosis*  *C. parapsilosis*  *C. glabrata*  *C. glabrata*  *C. glabrata*  *C. glabrata*  *C. glabrata*  *C. glabrata*  *C. glabrata*  *C. glabrata*  *C. glabrata*  *C. krusei* | 0.928  0.298  1.245  1.037  1.512  0.654  0.793  0.641  1.619  0.329  1.416  0.651  1.033  1.474  0.762  1.109  0.219  0.478  1.879  1.334  2.486  2.826  0.341  0.680  0.672  0.762  0.195  0.877  0.987  0.414  0.606  0.341  0.909  0.715  0.933  0.741  1.112  1.183  1.501  0.691  1.787  1.020  1.062  0.963  1.871  1.226  1.286  2.419  0.603  2.131  0.653  0.911  2.377  1.279  0.597  1.620  0.191  0.566  0.956  0.650  1.083  1.808  0.949  0.252  0.952  0.442  0.817  0.226 | 1.579  0.303  1.280  1.061  1.816  0.959  0.947  1.523  2.458  0.331  1.735  2.146  1.053  1.358  0.962  0.829  0.417  0.622  2.575  1.445  1.934  2.481  0.250  0.746  0.951  0.696  0.252  1.753  1.044  0.860  0.625  0.643  1.147  0.651  1.201  0.848  1.235  1.266  1.191  0.858  2.484  1.333  0.931  1.434  1.638  2.352  1.218  2.486  0.427  2.632  0.520  1.045  2.194  1.287  1.014  1.914  0.462  0.942  1.556  0.617  1.739  1.613  2.048  0.367  0.759  0.543  0.632  0.355 | 0.965  0.258  1.011  1.088  1.657  1.100  0.473  1.307  2.232  0.276  1.288  2.204  0.873  1.164  0.970  0.624  0.331  0.636  2.370  1.485  1.907  2.482  0.541  0.762  0.857  0.602  0.227  1.850  1.277  0.651  0.608  0.327  1.138  0.609  1.090  0.774  1.028  1.316  1.092  0.401  2.498  1.020  1.226  0.938  1.556  2.459  0.863  2.299  0.351  2.560  0.729  0.844  2.103  1.785  0.853  1.672  0.229  1.184  1.247  0.407  1.522  2.021  1.866  0.661  0.665  0.876  0.788  0.313 | 0.95  0.30  1.26  1.07  1.58  1.03  0.87  1.42  2.35  0.33  1.35  2.18  1.04  1.42  0.97  0.73  0.37  0.63  2.47  1.47  1.92  2.48  0.30  0.75  0.90  0.73  0.24  1.80  1.02  0.76  0.61  0.33  1.14  0.63  1.01  0.76  1.17  1.29  1.14  0.77  2.49  1.02  1.00  0.95  1.60  2.41  1.25  2.45  0.39  2.60  0.69  0.98  2.15  1.28  0.93  1.65  0.21  1.06  1.10  0.63  1.63  1.71  1.96  0.31  0.71  0.49  0.80  0.33 |

| No. of isolate | Species of *Candida* | Testing assay: | | | |
| --- | --- | --- | --- | --- | --- |
| Non-persistent group |  | Crystal violet | | | Average* |
| NA1  NA2  NA3  NA4  NA5  NA6  NA7  NA8  NA9  NA10  NA11  NA12  NA13  NA14  NA15  NA16  NA17  NA18  NA19  NA20  NA21  NA22  NA23  NA24  NA25  NA26  NA27  NA28  NA29  NA30  NA31  NA32  NA33  NT1  NT2  NT3  NT4  NT5  NT6  NT7  NT8  NT9  NT10  NT11  NT12  NT13  NT14  NT15  NT16  NT17  NP1  NP2  NP3  NP4  NP5  NP6  NP7  NP8  NG1  NG2  NG3  NG4  NG5  NG6  NG7  NG8  NG9  NK1 | *C. albicans*  *C. albicans*  *C. albicans*  *C. albicans*  *C. albicans*  *C. albicans*  *C. albicans*  *C. albicans*  *C. albicans*  *C. albicans*  *C. albicans*  *C. albicans*  *C. albicans*  *C. albicans*  *C. albicans*  *C. albicans*  *C. albicans*  *C. albicans*  *C. albicans*  *C. albicans*  *C. albicans*  *C. albicans*  *C. albicans*  *C. albicans*  *C. albicans*  *C. albicans*  *C. albicans*  *C. albicans*  *C. albicans*  *C. albicans*  *C. albicans*  *C. albicans*  *C. albicans*  *C. tropicalis*  *C. tropicalis*  *C. tropicalis*  *C. tropicalis*  *C. tropicalis*  *C. tropicalis*  *C. tropicalis*  *C. tropicalis*  *C. tropicalis*  *C. tropicalis*  *C. tropicalis*  *C. tropicalis*  *C. tropicalis*  *C. tropicalis*  *C. tropicalis*  *C. tropicalis*  *C. tropicalis*  *C. parapsilosis*  *C. parapsilosis*  *C. parapsilosis*  *C. parapsilosis*  *C. parapsilosis*  *C. parapsilosis*  *C. parapsilosis*  *C. parapsilosis*  *C. glabrata*  *C. glabrata*  *C. glabrata*  *C. glabrata*  *C. glabrata*  *C. glabrata*  *C. glabrata*  *C. glabrata*  *C. glabrata*  *C. krusei* | 0.805  0.527  0.634  0.630  0.358  1.183  0.270  0.211  0.255  0.780  1.109  1.016  0.582  0.256  0.126  0.184  0.895  0.716  0.598  0.386  0.358  0.673  0.243  0.146  0.388  0.613  0.721  0.223  0.353  1.017  0.521  0.255  0.569  0.830  1.631  0.778  0.755  1.074  0.805  1.284  1.153  1.711  0.881  0.317  0.878  0.317  0.354  0.733  0.516  0.265  2.203  0.262  3.037  0.523  0.651  2.594  0.411  0.351  0.347  0.383  0.266  0.304  0.371  0.349  0.296  0.572  0.336  0.336 | 0.688  0.655  0.875  0.842  0.295  1.197  0.228  0.309  0.292  0.839  0.714  0.808  0.476  0.181  0.164  0.168  1.173  0.666  0.859  0.485  0.372  0.617  0.172  0.164  0.464  0.808  0.754  0.281  0.446  1.217  0.385  0.241  0.578  0.828  1.202  1.101  0.788  1.145  0.701  1.041  1.156  1.740  1.001  0.183  1.117  0.344  0.338  0.950  0.672  0.321  2.509  0.370  2.484  0.323  0.775  2.018  0.142  0.372  0.370  0.481  0.569  0.483  0.251  0.408  0.319  0.752  0.320  0.364 | 0.711  0.575  0.902  0.758  0.212  1.279  0.234  0.247  0.337  0.783  1.001  0.737  0.693  0.198  0.132  0.195  0.947  0.617  1.042  0.569  0.702  0.601  0.194  0.154  0.564  0.926  0.739  0.359  0.572  1.092  0.808  0.382  0.515  0.420  0.837  1.282  0.822  1.389  0.825  1.022  1.018  1.217  1.053  0.169  0.990  0.330  0.466  0.738  0.489  0.340  3.135  0.243  2.777  0.340  0.467  2.294  0.647  0.266  0.408  0.377  0.448  0.436  0.310  0.457  0.598  0.892  0.354  0.415 | 0.70  0.55  0.89  0.80  0.33  1.19  0.23  0.23  0.27  0.78  1.06  0.77  0.53  0.19  0.13  0.19  0.92  0.64  0.95  0.53  0.37  0.61  0.18  0.15  0.43  0.87  0.75  0.25  0.40  1.05  0.45  0.25  0.57  0.83  1.02  1.19  0.77  1.11  0.82  1.03  1.15  1.73  1.03  0.18  0.93  0.32  0.35  0.74  0.50  0.33  2.36  0.25  2.91  0.33  0.71  2.16  0.53  0.36  0.36  0.38  0.51  0.46  0.34  0.43  0.31  0.82  0.33  0.35 |

* We abandoned the outlier and obtained the average according to the remaining 2 values.
